# Mechanical strategies to promote vascularization for tissue engineering and regenerative medicine

**DOI:** 10.1093/burnst/tkae039

**Published:** 2024-09-30

**Authors:** Yiran Wang, Meixuan Liu, Wei Zhang, Huan Liu, Fang Jin, Shulei Mao, Chunmao Han, Xingang Wang

**Affiliations:** Department of Burns and Wound Care Center, The Second Affiliated Hospital of Zhejiang University College of Medicine, 88 Jiefang Road, Shangcheng District, Hangzhou 310009, China; The Key Laboratory of the Diagnosis and Treatment of Severe Trauma and Burn of Zhejiang Province, 88 Jiefang Road, Shangcheng District, Hangzhou 310009, China; Department of Burns and Wound Care Center, The Second Affiliated Hospital of Zhejiang University College of Medicine, 88 Jiefang Road, Shangcheng District, Hangzhou 310009, China; The Key Laboratory of the Diagnosis and Treatment of Severe Trauma and Burn of Zhejiang Province, 88 Jiefang Road, Shangcheng District, Hangzhou 310009, China; Department of Burns and Wound Care Center, The Second Affiliated Hospital of Zhejiang University College of Medicine, 88 Jiefang Road, Shangcheng District, Hangzhou 310009, China; The Key Laboratory of the Diagnosis and Treatment of Severe Trauma and Burn of Zhejiang Province, 88 Jiefang Road, Shangcheng District, Hangzhou 310009, China; Department of Burns and Wound Care Center, The Second Affiliated Hospital of Zhejiang University College of Medicine, 88 Jiefang Road, Shangcheng District, Hangzhou 310009, China; The Key Laboratory of the Diagnosis and Treatment of Severe Trauma and Burn of Zhejiang Province, 88 Jiefang Road, Shangcheng District, Hangzhou 310009, China; Department of Burns and Wound Care Center, The Second Affiliated Hospital of Zhejiang University College of Medicine, 88 Jiefang Road, Shangcheng District, Hangzhou 310009, China; The Key Laboratory of the Diagnosis and Treatment of Severe Trauma and Burn of Zhejiang Province, 88 Jiefang Road, Shangcheng District, Hangzhou 310009, China; Department of Burns and Plastic Surgery, Quhua Hospital of Zhejiang, 62 Wenchang Road, Quhua, Quzhou 324004, China; Department of Burns and Wound Care Center, The Second Affiliated Hospital of Zhejiang University College of Medicine, 88 Jiefang Road, Shangcheng District, Hangzhou 310009, China; The Key Laboratory of the Diagnosis and Treatment of Severe Trauma and Burn of Zhejiang Province, 88 Jiefang Road, Shangcheng District, Hangzhou 310009, China; Department of Burns and Wound Care Center, The Second Affiliated Hospital of Zhejiang University College of Medicine, 88 Jiefang Road, Shangcheng District, Hangzhou 310009, China; The Key Laboratory of the Diagnosis and Treatment of Severe Trauma and Burn of Zhejiang Province, 88 Jiefang Road, Shangcheng District, Hangzhou 310009, China

**Keywords:** Vascularization, Tissue engineering, Mechanical cues, Forces, Prevascularized tissue, Regeneration, Scaffold, Stretch

## Abstract

Vascularization is a major challenge in the field of tissue engineering and regenerative medicine. Mechanical factors have been demonstrated to play a fundamental role in vasculogenesis and angiogenesis and can affect the architecture of the generated vascular network. Through the regulation of mechanical factors in engineered tissues, various mechanical strategies can be used to optimize the preformed vascular network and promote its rapid integration with host vessels. Optimization of the mechanical properties of scaffolds, including controlling scaffold stiffness, increasing surface roughness and anisotropic structure, and designing interconnected, hierarchical pore structures, is beneficial for the *in vitro* formation of vascular networks and the ingrowth of host blood vessels. The incorporation of hollow channels into scaffolds promotes the formation of patterned vascular networks. Dynamic stretching and perfusion can facilitate the formation and maturation of preformed vascular networks *in vitro*. Several indirect mechanical strategies provide sustained mechanical stimulation to engineered tissues *in vivo*, which further promotes the vascularization of implants within the body. Additionally, stiffness gradients, anisotropic substrates and hollow channels in scaffolds, as well as external cyclic stretch, boundary constraints and dynamic flow culture, can effectively regulate the alignment of vascular networks, thereby promoting better integration of prevascularized engineered tissues with host blood vessels. This review summarizes the influence and contribution of both scaffold-based and external stimulus-based mechanical strategies for vascularization in tissue engineering and elucidates the underlying mechanisms involved.

Highlights

- Mechanical factors play a crucial role in pre-vascularization
- Mechanical properties of scaffolds, including stiffness, surface roughness, structure anisotropy and pore size regulate pre-vascularization and the ingrowth of host blood vessels.
- Incorporating hollow channels into scaffolds promotes the formation of patterned vascular networks.
- Dynamic stretching and perfusion culture facilitate the formation and maturation of *in vitro* pre-formed vascular networks.
- Mechanical factors regulate the alignment of vascular networks, thereby promoting better integration of pre-vascularized engineered tissues with host blood vessels.

## Background

Since the introduction of the tissue engineering field by Langer and Vacanti in 1993, great progress has been made in both tissue engineering and regenerative medicine, with applications in numerous tissues and organs (e.g. skin, trachea, bladder, urethra, myocardial tissue and periodontal tissue) [1, 2]. Currently, vascularization is one of the primary hurdles in tissue engineering, especially for thick and complex structures. After implantation, engineered tissues require an adequate vascular network to provide oxygen and nutrients to support cell survival and regeneration [3].

Physiologically, vascularization can be separated into two processes: vasculogenesis, which relies on the differentiation and proliferation of angiogenic or progenitor cells within avascular tissues to form a primitive capillary network, and angiogenesis, which is the formation of new blood vessels, through sprouting, from the existing vascular network [4–6]. Traditionally, vascularization in engineered grafts has been achieved through the ingrowth of angiogenic sprouts from surrounding host tissues. However, this process is limited by the speed of angiogenesis, as the average rate for individual vessels is ~5 μm/h [7, 8]. Although strategies such as the addition of angiogenic factors, optimization of scaffolds and use of gene delivery can effectively accelerate angiogenesis, the vascularization interval for larger tissue grafts is still too long to meet survival needs after implantation [9, 10]. To further shorten the vascularization time of tissue-engineered constructs, researchers have proposed the concept of prevascularization before implantation [11–13]. The advantage of prevascularization lies in the rapid perfusion of tissue grafts through the preformed microvascular network connected to the host vasculature. However, prevascularization still faces the challenges of maintaining the stability and maturity of preexisting vascular structures and integrating with host vasculature, among others [8, 14, 15].

The integration of a dense, full-range, mature and functional vascular network within scaffolds, which can rapidly integrate with the host vasculature, seems to be the ideal solution to address the abovementioned challenges [8, 16]. Specifically, first and foremost, to meet the oxygen and nutrient requirements of seeded cells in engineered tissue, the preformed network must achieve full tissue coverage with a spacing of <20 μm. Second, the network should be sufficiently mature and stable to ensure blood perfusion without excessive leakage. Third, to maintain sufficient blood flow, the network must exhibit a hierarchical structure from large arteries to small capillaries [17]. Fourth, the vascular network must function to exchange substances; thus, the end branches of the network should consist of a monolayer of endothelial cells (ECs) with a very high level of cellular activity. Finally, the preformed vessel network needs to integrate quickly with the host blood vessels, which depends on the degree of orientation and morphological matching between the preformed vascular network and the mature host blood vessels at the implantation sites [18–20].

**Figure 1:**
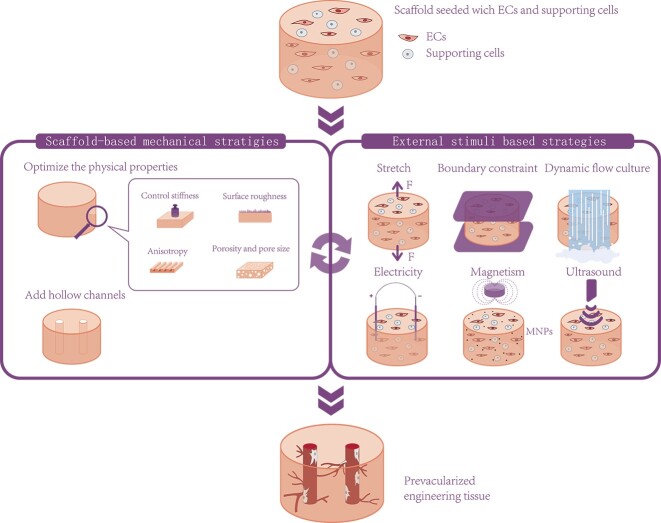
Mechanical strategies to promote vascularization for tissue engineering. *ECs* endothelial cells, *MNPs* magnetic nanoparticles

*In vivo*, the vascular network is exposed to a complex mechanical environment composed of both extrinsic stresses induced by blood vessel haemodynamic stress and intrinsic stresses induced by cellular traction of cell–cell and cell–extracellular matrix (ECM) adhesions. The mechanical environment regulates the structure and function of the vascular network as well as the rate and direction of angiogenesis [21–24]. The primary objective of tissue engineering is to mimic the *in vivo* environment *in vitro* to optimize cell growth and tissue formation. Therefore, simulating the mechanical environment to promote angiogenesis *in vivo* (e.g. stiffness of the ECM and shear forces due to blood flow) provides clear directions for the design of mechanical strategies that promote vascularization for tissue engineering [3, 25]. Currently, the application of various strategies to improve the mechanical environment has been shown to be effective in promoting the process of vascularization, particularly in optimizing the preformed vessel network *in vitro*. In this review, we summarize the influence and contribution of both scaffold-based mechanical strategies and external mechanical stimuli-based strategies in tissue engineering vascularization and elucidate the mechanisms involved (Figure 1).

## Review

### Prevascularization and the effects of mechanical strategies

Prevascularization refers to the addition of preformed vascular networks before implantation to reduce the time and complexity of *in vitro* vessel formation [11, 14, 15]. The cells involved in prevascularization can be categorized into cells of an endothelial lineage and supporting cells. ECs are the main cells that compose the inner wall of blood vessels, and various types of endothelial lineage cells are used to construct preformed vascular networks. Among them, human umbilical vein ECs (HUVECs) are widely used in coculture systems due to their ease of isolation and low cost [26]. However, terminally differentiated ECs have limited proliferation capacity, which restricts their potential application in prevascularization strategies. Stem cells with self-renewal and directional differentiation capabilities have received increased attention as seed cells for prevascularization. Embryonic stem cells, bone marrow-derived mesenchymal stem cells, human induced pluripotent stem cells and endothelial progenitor cells have all been shown to differentiate into ECs in endothelial differentiation media supplemented with growth factors [26, 27]. Supporting cells are cells that grow with ECs and play a supportive and stabilizing role in newly formed blood vessels. Supporting cells include fibroblasts, smooth muscle cells, pericytes and osteoblasts, among others. In addition to providing structural support, these cells also secrete angiogenic factors, such as vascular endothelial growth factor (VEGF) and basic fibroblast growth factor, and regulate the process of vascularization through paracrine effects [11, 28–30].

A simple method to achieve prevascularization is the co-seeding of endothelial lineage cells and supporting cells within a suitable scaffold. Through proliferation, migration and connection, ECs self-assemble to form capillary-like structures and recruit supporting cells to stabilize the scaffolds [11–13]. In addition to the implantation of individual cells, some researchers achieve prevascularization by direct implantation of microvascular fragments or intact microvessels into scaffolds [8, 14, 15]. Moreover, patterned vascular networks can be achieved by integration of hollow channels within scaffolds, which will be described in detail later in this manuscript.

Mechanical factors play a crucial role in vasculogenesis and angiogenesis and can affect the architecture of the generated vascular network [21–24]. The formation of vascular networks *in vitro* is more dependent on the regulation of various mechanical factors due to the loss of the complex mechanical environment present *in vivo*. Mechanical strategies aimed at promoting prevascularization involve the modulation of various mechanical factors, which activate endothelial progenitor cells and supporting cells *in vitro* through mechanical pathways, thereby enhancing the formation and maturation of vascular networks and controlling their alignment. Among them, scaffold-based strategies optimize the cell–ECM and cell–cell interaction forces, whereas external mechanical strategies provide direct mechanical stimulation. Table 1 summarizes the mechanical strategies used to control the orientation of the preformed vascular network and its alignment directions [19, 31–37].

**Table 1:** Mechanical strategies to control the orientation of vascular network

	**Strategies**	**Main mechanism**	**Alignment direction**	**Example reference**
**Scaffold-based**	Stiffness gradients	Cellular traction force	Parallel to the direction of stiffness gradients	[31]
	Anisotropic substrate	Contact guidance	Consistent with the grooves or aligned fibres	[32]
	Hollow channels	Contact guidance	Consistent with hollow channels	[33, 34]
**External stimuli**	Cyclic stretch	Stretch force	Orthogonal to the direction of resultant force (stretch force and passive compressive strain)	[19, 35]
	Boundary constraint	Reaction of cellular traction force	Parallel to the constraint axis	[19, 36]
	Dynamic flow culture	Shear stress	Parallel to the direction of shear stress	[37]

### Mechanical strategies based on scaffolds

As cell carriers in tissue engineering, scaffolds provide a temporary microenvironment for cell adhesion, proliferation, differentiation, communication and junctions and serve as a guide for the formation of new tissues and organs [38]. Cells can sense various mechanical cues in scaffolds (e.g. substrate stiffness, substrate topography and spatial structure) and react via biochemical mediators such as the cytoskeleton [39, 40]. In recent years, researchers have optimized these mechanical cues by adjusting the material and structure of the scaffolds, which has markedly increased their vascularization [25, 41–45].

#### Optimizing the physical properties of scaffolds

##### Stiffness

Substrate stiffness is an important mechanical parameter that affects cell function and fate [39, 46]. Many studies have investigated the structural and functional changes in ECs and pericytes cultured *in vitro* on substrates with different stiffnesses [47–49]. The results indicate that within a certain range, higher substrate stiffness promotes EC adhesion, proliferation and migration, as well as capillary-like tube formation and sprouting [50–54]. Nevertheless, in excessively stiff substrates, the proliferation of ECs may reach a plateau [50]. High EC traction force, secondary to high substrate stiffness, leads to loosened cell–cell junctions and a smaller substrate adhesion area, which contributes to disruption of the monolayer vascular network and a reduction in collective migration [48]. Moreover, increased substrate stiffness is related to increased inflammatory gene expression, increased monocyte adhesion and inhibition of nitric oxide expression, which may lead to the dysfunction of ECs and supporting cells and long-term adverse effects after scaffold implantation [55–57]. A recent study showed that ECs within a stiff ECM induce pericyte–myofibroblast differentiation through paracrine pathways, thereby affecting the stability and function of microvessels [57]. Appropriate substrate stiffness can also promote vasculogenesis by facilitating the differentiation of stem cells and endothelial progenitor cells [58, 59]. Another recent study demonstrated that medium-stiffness substrates, compared with both stiff and soft substrates, enhance the differentiation of induced pluripotent stem cells into heart valve ECs [59].

**Figure 2:**
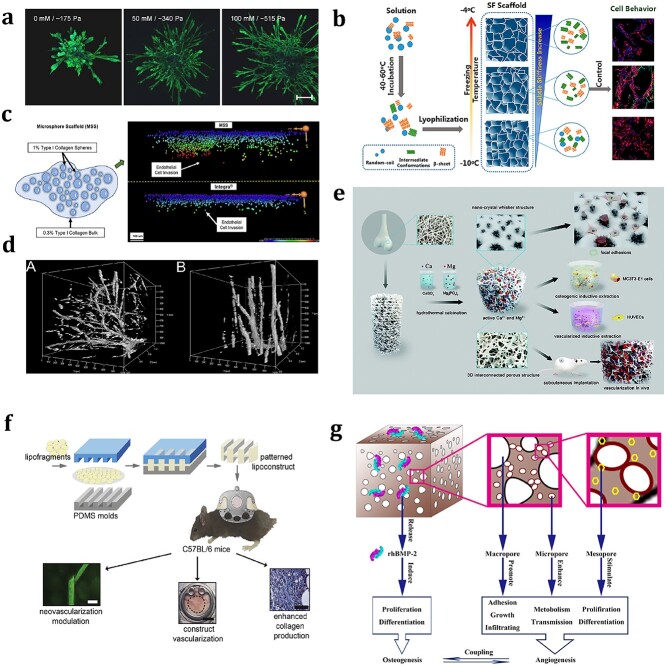
Optimization of the physical properties of scaffolds to promote vascularization. (**a**) EC spheroids were embedded in collagen hydrogels with different matrix stiffnesses (0.175, 0.340 and 0.515 kPa), which were independent of collagen concentration and architecture. With increasing matrix stiffness, the number and length of angiogenic sprouts and overall spheroid outgrowth significantly increased. [25] (Copyright 2012 Acta Materialia Inc. Published by Elsevier Ltd.) (**b**) Silk fibroin scaffolds with refined control of stiffness (3.0–7.4, 13.0–25.9 and 35.6–58.4 kPa) were formed by introducing freezing temperature as a tunable regulating factor during the freeze-drying process. The fine regulation of stiffness was correlated with the vascularization capacity of these scaffolds. Reprinted from Lu *et al*. [41] (Copyright 2019 American Chemical Society) (**c**) Scaffolds with differential density interfaces were fabricated by encasing 1% type 1 collagen microspheres with a diameter of 50–150 μm in 0.3% collagen bulks. Compared with a similar product from Integra®, these scaffolds exhibited increased cellular invasion and neovascularization. Reprinted from Celie *et al*. (Copyright 2019 Acta Materialia Inc. Published by Elsevier Ltd.) (**d**) ECM and smooth muscle cell aggregates were seeded in a bulk polymerized hydrogel (homogeneous, 2.13 kPa) (A) and a diacrylate hydrogel with a stiffness gradient (3c2 to 0.62 kPa in the *y*-direction) (B). Aggregate invasion in the gradient hydrogel occurred bidirectionally and sprout alignment was observed in the direction parallel to the gradient while the bulk hydrogel resulted in uniform invasion. Reprinted from Turturro*et al*. [31] (Copyright 2013 Turturro et al.) (**e**) Degradable macroporous scaffold with novel nano-crystal whisker-like microstructures on the surface was developed using a hydrothermal calcination process. Both *in vitro* and *in vivo* assays demonstrated that the 3D interconnected porous architecture with nano-crystal surface microstructure promoted vascularization and tissue integration. Reprinted from Chu *et al*. [62] (Copyright 2018 The Royal Society of Chemistry) (**f**) Surface-structured lipofragments were formed with different polydimethylsiloxane moulds as prevascularized scaffolds and implanted into the dorsal skinfold chamber of mice. The lipofragments with a 5 μm groove depth exhibited faster vascularization, a stabilized microvessel network, less inflammation and greater collagen deposition. Reprinted from McLuckie *et al*. [63] (Copyright 2020 Acta Materialia Inc. Published by Elsevier Ltd.) (**g**) Hierarchical pore structure of the scaffolds facilitated vascularization. Micropores (20–50 μm) enhanced porosity and promoted metabolic transmission, mesopores stimulated cell proliferation and differentiation, and macropores (300–500 μm) promoted cell adhesion, growth and infiltration. Reprinted from Liu *et al*. [45] (Copyright 2020 Elsevier B.V.) *ECs* endothelial cells, *SF* silk fibroin, *MSS* microsphere scaffold, *HUVECs* Human umbilical vein ECs, *PDMS* polydimethylsiloxane, *rhBMP-2* recombinant human bone morphogenetic protein-2

In tissue engineering, the stiffness of the substrate exposed to the cells in the scaffold is mainly affected by the stiffness of the scaffold material, which can be tuned in various ways (e.g. by utilizing various cross-linking techniques, manipulating the chemical composition of the scaffold materials or modifying the scaffold architecture) [39, 49]. Mason *et al*. [25] tuned the stiffness of collagen-based scaffolds without significant architectural changes by glycating the collagen in solution, and the angiogenic outgrowth of embedded EC spheroids was significantly enhanced by increasing the scaffold stiffness (from 0.175 to 0.515 kPa) (Figure 2a). Lu *et al*. [41] introduced freezing temperature as an adjustable factor in the freeze-drying process to produce silk fibroin scaffolds with fine-tuning of stiffness in three different ranges (3.0–7.4, 13.0–25.9 and 35.6–58.4 kPa). By comparing the vascularization outcomes of scaffolds with different levels of stiffness adjustment, they identified the optimal stiffness for vascularization (a scaffold stiffness of 5.7 kPa). This scaffold stiffness was confirmed *in vitro* to promote the differentiation of bone marrow mesenchymal stem cells into ECs, the rapid migration of ECs and vessel structure formation, as well as the ingrowth of new blood vessels after implantation *in vivo* (Figure 2b). Overall, vascularization in tissue engineering relies on appropriate scaffold stiffness, as scaffolds that are too soft or too hard can negatively impact vasculogenesis and angiogenesis. However, the optimal stiffness for the promotion of vascularization in engineered tissue constructs may vary with the material and structure of the scaffold, the types of ECs and supporting cells, and other factors [60, 61]. During the process of designing the scaffolds, the stiffness can be finely adjusted through various means and determining the most suitable stiffness for vascularization based on further experimental results may be an effective strategy.

When the substrate stiffness is inhomogeneous, cells will migrate towards the areas with optimal stiffness for the generation of maximal cell traction force [49,64]. Using this principle, Celie *et al*. [42] prepared special microarchitecture scaffolds with differential densities by embedding high-density type I collagen microspheres in a low-density collagen bulk. *In vivo*, these scaffolds exhibited significantly greater cellular invasion and new blood vessel formation that spanned the entire scaffold depth (Figure 2c). Additionally, the formation of stiffness gradients in scaffolds can be used to regulate vascular orientation. Using perfusion-based frontal photopolymerization, Turturro*et al*. [31] formed a polyethylene glycol diacrylate hydrogel and decreased the elastic modulus from 3.2 to 0.62 kPa over a span of 10 mm. After seeding coculture aggregates of ECs and smooth muscle cells for 3 weeks, the hydrogels with a stiffness gradient demonstrated sprout lengths that were twice as long in the direction parallel to the gradient as those in the direction perpendicular to the gradient (Figure 2d).

##### Surface roughness

Increased surface roughness can promote integrin expression and enhance cell adhesion, growth and elongation [60,65]. Mokhtari and Zargar [44] created a poly(glycerol sebacate)/poly(lactic acid) composite scaffold by an electrospinning method and modified the scaffold with oxygen plasma for use as a vascular graft. Plasma surface modification successfully increased the roughness of the scaffold and did not induce adverse effects on its mechanical properties, degradation rate or haemocompatibility. Compared with the unmodified scaffold, the modified scaffold significantly improved the adhesion, growth and proliferation of HUVECs *in vitro*. Similarly, by using a volatile chemical to trigger phase separation during the electrospinning process, Han *et al*. [66] prepared a novel electrospun fibre scaffold with surface dimples. This unique scaffold improved the cellular adhesion and elongation of HUVECs and resulted in the development of an EC-based prevasculature *in vitro*. In another study, Chu *et al*. [62] used a hydrothermal calcination method to convert bovine cancellous bone into a degradable macroporous scaffold with a nanocrystal surface microstructure. This microstructure significantly promoted the gene expression of integrin and vinculin, thus facilitating the spread, adhesion and proliferation of cells. Both *in vitro* and *in vivo* assays demonstrated that these special microstructures with macroporous surfaces promoted highly effective osteogenesis and vascularization (Figure 2e). The above studies indicate that increasing the surface roughness or internal pores of the scaffold is an effective strategy for promoting vascularization in tissue engineering.

##### Anisotropic structures

Compared with cells on isotropic substrates, cells on anisotropic substrates seem to have stronger adhesion and oriented migration capabilities [60]. Therefore, scaffolds with anisotropic structures seem to be more conducive to the formation and ingrowth of their internal microvascular network. McLuckie *et al*. [63] prepared lipofragments with different polydimethylsiloxane moulds (flat, 5-μm and 50-μm parallel gratings) and implanted them in a dorsal skinfold chamber mouse model. A comparison of three lipoconstructs with different surface topographies revealed that the anisotropic topographical structures generated by the polydimethylsiloxane mould with 5-μm grating resulted in faster neovascularization and subsequent collagen production (Figure 2f). In fibrous scaffolds, proper fibre crimping mimics the anisotropic microstructure and mechanics of native substrates and alters the strain transmission of cells [67]. Davidson *et al*. [43] established a crimped fibrous scaffold using a new and simple method for engineering electrospun fibrous matrices composed of dextran vinyl sulfone with a controllable crimped structure. The matrices that possessed crimped fibres induced pronounced changes in mechanical nonlinearity, which led to an increased migration speed and promotion of rapidly assembled capillary-like networks. In addition, through contact guidance, highly anisotropic substrates with grooves or aligned fibres induce the elongation and alignment of ECs and increase the directionality of migration in favour of controlling vascular orientation [60, 68, 69]. By imposing external geometrical confinement with a large aspect ratio, Casale *et al*. [32] developed a soft connective tissue with aligned collagen fibres. After EC seeding, these anisotropic fibres guided the morphogenesis of a highly aligned capillary-like network.

##### Porosity and pore size

The 3D spatial structure guides the organization and orientation of microvascular networks through geometric constraints [70]. As important parameters of 3D scaffolds, porosity and pore size significantly impact the vascularization of engineered tissues. Successful vascularization requires a relatively large pore size (~100 μm) and sufficient pore interconnectivity to provide space; however, excessively large pores (>200 μm) and insufficient support structures may lead to a low-density vascular network [71–74]. Arnal-Pastor *et al*. [75] cocultured HUVECs and adipose tissue-derived stem cells in two scaffolds with different pore structures. One consisted of isotropically intersecting spherical (sponge-like) pores ~90 μm in diameter, while the other was composed of orthogonally intersecting arrays of cylindrical channels (grid-like) with a diameter of ~150 μm. The authors found that the former structure had wider pores and greater pore interconnectivity, which provided the necessary freedom for ECs to reorganize and form branched cell networks; this structure also promoted greater cell organization into tubular-like structures.

In contrast to single pore sizes, hierarchical porous structures seem more conducive to the formation of hierarchical vascular branching networks and the regulation of other cells in engineered tissues [76–78]. Liu *et al*. [45] fabricated hierarchical porous structured scaffolds based on poly(3-hydroxybutyrate-co-3-hydroxyhexanoate) containing micropores (20–50 μm), mesopores and macropores (300–500 μm). The micropores enhanced the porosity of the scaffolds and promoted metabolic transmission, the mesopores stimulated cell proliferation and differentiation, and the macropores promoted cell adhesion, growth and infiltration. *In vitro* seeding of HUVECs on these scaffolds demonstrated that the unique scaffold structure could rapidly and effectively induce the formation of vascular networks. When applied to the repair of critical-sized bone defects in rabbits, scaffolds with multilevel pores effectively promoted vascularization and enhanced bone regeneration [45] (Figure 2g).

Overall, the various physical properties of scaffolds have a significant impact on vascularization. Among them, the stiffness gradient and the highly anisotropic substrates within the scaffold may effectively control the orientation of the vascular network and its alignment directions. Optimal scaffold stiffness, greater surface roughness, anisotropic structure and interconnected, hierarchical pore structures with sufficient space are conducive to better vascularization in tissue engineering. However, the promotion of vascularization by optimizing the physical properties of scaffolds has some limitations. First, changing the physical performance of a single scaffold remains challenging, as changes are typically coupled with the other physical and chemical properties of the scaffold [39, 49]. For example, when the scaffold stiffness is changed by manipulating its chemical composition or modifying the scaffold architecture, the chemical and structural properties of the scaffold might also be altered [61]. In addition, the physical properties of the scaffold must be suitable for other cells within the scaffold and adapted to the target tissue after implantation. Blindly pursuing physical properties suitable for vascularization may lead to impairment of other functions of the scaffold. For example, increased surface roughness may increase the inflammatory response following implantation, which would intensify the foreign body response and potentially hinder tissue repair and regeneration [79]. Moreover, it is worth noting that the physical properties of the scaffold change dynamically due to the dynamic interaction forces exerted by the cells within the scaffold, as well as various external forces applied post-implantation [39]. When designing the scaffold, in addition to the properties mentioned above, the viscoelasticity, viscoplasticity and biodegradability of materials are equally essential [80, 81].

#### Addition of hollow channels and patterned microvasculature

**Figure 3:**
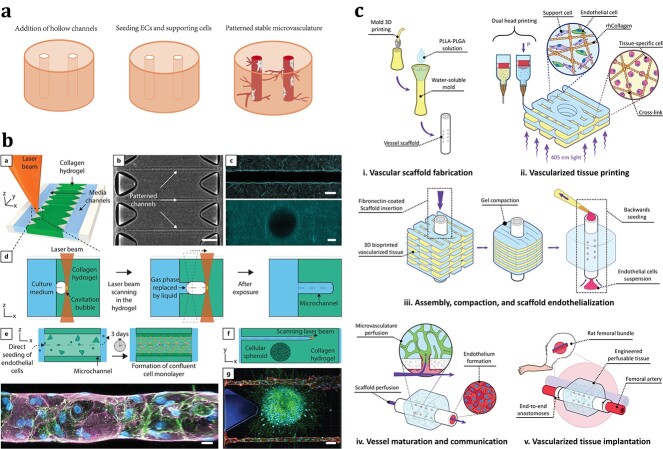
Preformed patterned microvasculature construction. (**a**) Schematic diagram of the process of constructing patterned microvasculature in tissue engineering. (**b**) Collagen hydrogels were patterned by femtosecond laser irradiation to create hollow channels with diameters as small as 20 μm. The channels enabled the formation of artificial microvasculature by the culture of ECs and cell media perfusion and preserved the viability of cell spheroids encapsulated in the hydrogels either as single cells or spheroids. Reprinted from Enrico *et al*. [34] (Copyright 2022 The Authors. Advanced Materials published by Wiley-VCH GmbH) (**c**) Millimetric vessel-like scaffold and 3D bioprinted vascularized tissue were fabricated and interconnected to create an engineered hierarchical vascular construct. The vascular construct was successfully perfused with host blood *in vivo*. Reprinted from Szklanny *et al*. [17] (Copyright 2021 The Authors. Advanced Materials published by Wiley-VCH GmbH) *ECs* endothelial cells, *PLLA* poly(l-lactic acid), *PLGA* poly(lactic-co-glycolic acid), *rhCollMA* recombinant human collagen methacrylate

The addition of hollow channels within the scaffold not only helps to transfer oxygen and nutrients but also provides specialized curved topographical cues for cells within the scaffold [82, 83]. The curved topography imposes lateral constraints on the growth of focal adhesions and actin stress fibres, thereby driving anisotropic forces and inducing cell alignment and migration along the direction of the channel [60, 84]. After implantation of scaffolds with hollow channels into a host, the growth of host blood vessels in the direction of the channels can increase, thereby accelerating the process of vascularization [85]. *In vitro*, when endothelial lineage cells and supporting cells are seeded into a scaffold with hollow channels, localized and confluent endothelialization can occur on the channel walls, while supporting cells are recruited to the outer side of the channels, where they eventually form vascular structures consistent with the channel structure [85, 86] (Figure 3a). Zieber *et al*. [33] used a carbon dioxide laser engraving system to form 200-μm-diameter channels from the top to the bottom of a 2-mm-thick alginate scaffold. HUVECs, cardiomyocytes and cardiofibroblasts were then seeded onto the scaffolds in that order. Cardiac cell constructs with stable vessel-like networks were formed, wherein the HUVECs became organized around the channels in a multilayer manner, while the cardiomyocytes were localized between the channels and exhibited the characteristic morphological features of mature cardiac fibres. In recent years, patterned microvasculature has been introduced for tissue engineering by inserting predefined hollow channels into scaffolds and by seeding or perfusing ECs directly into the channels [34, 87]. Compared with a self-assembled microvascular network, a patterned microvasculature is controllable and reproducible and facilitates the formation of tissue-specific hierarchical vascular structures [8, 88, 89].

However, a patterned microvasculature with the structure and function of native capillaries is difficult to achieve. Due to technical limitations, the diameter of a patterned microvasculature is usually larger than that of a physical microvascular network. Conventional bioprinting only allows the fabrication of hydrogel scaffolds containing vessel-like hollow channels with diameters >100 μm, while the average capillary diameter is only 6–9 μm [34, 90]. After perfusion, a patterned microvasculature of relatively large diameter will exert forces different from those of the native microvasculature and induce changes in EC function that are not conducive to oxygen and nutrient exchange [34, 91]. In addition, the branching structures and network density are also limited by technology, which increases the difficulty in achieving a tissue-wide oxygen supply [92, 93]. Table 2 shows a comparison between self-assembled microvasculature and patterned microvasculature.

**Table 2:** Comparison of self-assembled microvasculature and patterned microvasculature in tissue engineering

	**Self-assembled microvasculature**	**Patterned microvasculature**
**Mode of formation**	Co-seed endothelial cells and supporting cells within a suitable environment to enable spontaneous organization and further angiogenesis into microvascular networks	Directly seed or perfuse ECs into the surface of pre-defined patterned channels to form endothelialized vessel structures. The endothelialized vessel structures further recruit supporting cells to form stable vessels
**Features of structure**	Random, heterogeneous, complex	Controllable, simple
**Main mechanical factors**	1. Stiffness and topography of substrate 2. External stretch	1. Diameter and pattern of channels 2. Flow and shear stress
**Advantages**	Better mimic the structure and function of natural capillaries	1. Speed-up the vascularization process 2. Precisely control the size and orientation of vessels
**Limitations**	1. The process is relatively slow 2. Lack of controllability and replicability 3. Difficult to form hierarchical structures and integrate host vessels	1. The diameter of microvessels was limited which affects the functions of endothelial cells and is detrimental to nutrient exchange 2. Difficult to achieve the ideal branching structures and network density 3. Require high technical and high equipment support

With continuous research and technological advances, the limitations of patterned microvasculature have gradually been overcome. The shape and branching of the channels have become controllable. Using a gelatine/bacterial cellulose hydrogel and chitosan-gelatine/β-glycerophosphate ink hydrogel, Hu *et al*. [15] printed hydrogel scaffolds with various prevascularized channels, including circular, branched and tree-shaped networks, to confirm a high degree of freedom in the geometry. The diameter of this microvasculature approached that of native capillary blood vessels. Using high-resolution electrohydrodynamic inkjet printing with a sacrificial Pluronic F-127 solution, Zheng *et al*. [92] fabricated hydrogel-based microvascular tissues exhibiting hierarchical and branching channels with a minimum feature size of 30 μm. Using laser-based cavitation moulding technology, Enrico *et al*. [34] fabricated geometrically controlled 3D microchannels with diameters as small as 20 μm without affecting the viability outside the channels. These microchannels enabled the formation of artificial microvasculature by EC culture and cell media perfusion (Figure 3b). Combinations of self-assembled and patterned formation techniques have been used to construct hierarchical vessel networks [17, 18]. Szklanny *et al*. [17] used collagen bioink containing human adipose microvascular ECs (HAMECs) and dental pulp stem cells to manufacture bioprinted tissue with microvasculature. The bioprinted tissue was then assembled with an endothelial vessel-like scaffold. When the ends of the vessel-like scaffold were directly anastomosed with the host artery, the hierarchical vascular tissues preformed in combination were successfully perfused by host blood (Figure 3c). Son *et al*. [88] bioprinted perfusable endothelialized channels and connected them with a specific bridge pattern. Through the spatial gradient of angiogenic factors that the authors had designed, the capillary networks grew along the bridge pattern and connected the endothelialized channels. These capillary networks with a controllable architecture were completely formed by angiogenesis. Overall, the incorporation of hollow channels into scaffolds to create a patterned microvascular network is a promising approach for prevascularization in tissue engineering. In the future, continuous technological innovations will still be needed to construct hollow channel structures similar to hierarchical microvasculature *in vivo* to optimize the implantation methods of cells and to develop better *in vitro* cultivation methods after implantation.

### Mechanical strategies based on external stimuli

The vasculature is continuously subjected to mechanical stimuli due to dynamic activities such as blood flow and muscle contractions. These mechanical stimuli elicit responses by ECs, pericytes, smooth muscle cells and other cells, which ultimately influences the structure and function of the vasculature. In tissue engineering, mechanical strategies based on external stimuli can be used to mimic the mechanical stimuli present in the body to a certain extent, thereby promoting vascularization [19, 60, 94]. Mechanical strategies based on external stimuli can be categorized into direct mechanical strategies and indirect mechanical strategies. Direct mechanical strategies, which involve the direct application of forces to tissue-engineered scaffolds, are primarily used for the *in vitro* cultivation of tissue-engineered constructs and can effectively regulate the formation and alignment of preformed vascular networks. Indirect mechanical strategies involve the exertion of mechanical effects on tissue-engineered structures without direct contact, not only for *in vitro* cultivation but also for further optimization after implantation *in vivo*.

#### Direct external mechanical strategies

##### Stretch and boundary constraints

Since pulsatile blood pressure transmits tensile stress to blood vessel walls, ECs and perivascular cells are continuously subjected to cyclic stretch force (5–10% the magnitude of mechanical stretch) *in vivo* [95, 96]. The stretch force is transmitted from the ECM to the intracellular actin filament system via integrins and further activates endogenous biochemical cues [97]. A distinct cell response under stretch forces is the reorganization of actin stress fibres and focal adhesions, which induce cell elongation and uniform reorientation. *In vitro* studies have shown that the cell reorientation angle is generally orthogonal to the direction of the cyclic stretch force to maintain intracellular tension at an optimal level, and the speed of reorientation is related to the substrate properties and the strength and frequency of stretch [60, 98, 99]. In addition, the exposure of ECs and supporting cells to cyclic tensile strain upregulates the secretion of a variety of bioactive molecules, including angiopoietin 1, angiopoietin 2, platelet-derived growth factor β, VEGF and matrix metalloproteinase 2. These molecules play key roles in promoting the formation, sprouting, maturation and stabilization of vessel networks [35, 100–102].

**Figure 4:**
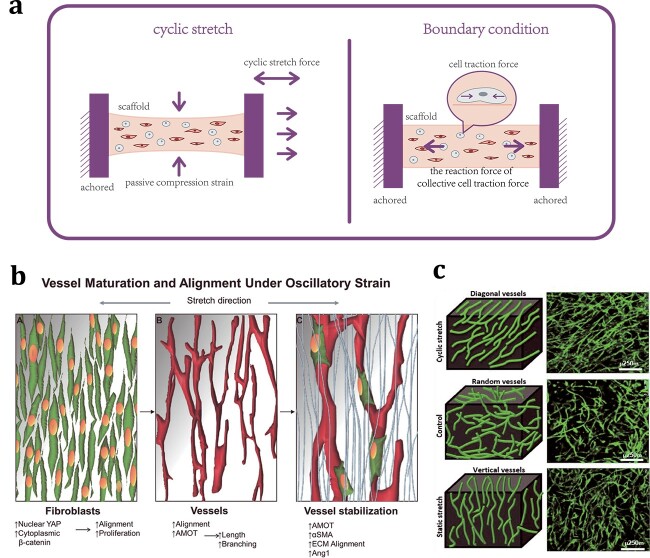
Application of stretching and boundary constraints to promote vascularization. (**a**) Schematic diagram of the application of cyclic stretching and boundary constraints on tissue-engineered constructs. (**b**) Application of cyclic stretch on a scaffold containing ECs cocultured with fibroblasts promoted the stabilization and alignment of neovessels. Cyclic stretch first induced the alignment and increased the proliferation of fibroblasts (green cells). Next, ECs formed aligned vessels according to fibroblast alignment and were more complex and longer than those of the static control. Fibroblasts were subsequently recruited to the vessels, and the ECM aligned and stabilized the vessels (light blue fibres). Reprinted from Landau *et al*. [35] (Copyright 2018 The Authors. Published by WILEY-VCH Verlag GmbH & Co. KGaA, Weinheim) (**c**) Application of cyclic stretch on a scaffold containing ECs cocultured with fibroblasts resulted in diagonal vessels, whereas static stretching resulted in vertical vessels, and free-floating resulted in randomly orientated vessels (green fluorescence: ECs). Reprinted from Rosenfeld *et al*. [19]. *ECs* endothelial cells, *ECM* extracellular matrix, *YAP* Yes-Associated Protein, *AMOT* Angiomotin, *αSMA* α-smooth muscle actin, *ECM* cell–extracellular matrix, *Ang1* Angiopoietin1

The application of tensile force to engineered tissues mimics the stretching stress induced by blood pressure fluctuations to some extent, which effectively promotes angiogenic responses and vessel alignment. Landau *et al*. [35] co-seeded HAMECs and fibroblasts on gel-foam scaffolds and placed them in a bioreactor to mimic uniaxial oscillatory stretching (20% strain, 1 Hz). Compared with the static control, the cyclically stretched vessel network developed a more elongated and branched structure. The stability of the cyclically stretched vessel network was enhanced through increased secretion of proangiogenic cytokines, mural cell differentiation and collagen VI deposition. Moreover, the cyclically stretched vessels were significantly oriented, which was shown to be mediated by the alignment of fibroblasts under oscillatory stretch (Figure 4b). Notably, in their study, the orientation of the vessels was basically perpendicular to the stretch direction [35]. However, using a similar construct and condition (10% strain, 1 Hz), Rosenfeld *et al*. [19] reported that oscillatory stretching resulted in diagonal vessel alignment, which was symmetrically organized 30–60° to the stretching direction (Figure 4c). The orientation direction induced by cyclic stretching may be orthogonal to the resultant force of tensile strain and passive compressive strain resulting from stretching, which is different under different stretch amplitudes and scaffold physical properties [60, 103] (Figure 4a).

Static tensile force similarly promotes angiogenic responses and vascular alignment [19, 36, 104, 105]. Zheng *et al*. [106] exposed coronary microvascular ECs to cyclic stretch (10% strain, 0.5 Hz) and static stretch (10% strain) in 2D culture conditions. Both types of stretch stimulation activate VEGF receptor 2 signalling and increase cell proliferation and tube formation, although cyclic stretch results in greater tube length and branch formation. Boundary constraint is a simple technique involving the application of static tensile forces to scaffolds. When no external load is applied, the cellular traction force generated by the cells inside makes the scaffolds, especially collagen gel-based scaffolds, prone to deformation and compaction [39, 107]. Using boundary constraints to anchor both ends of the scaffolds leads to a cell-induced collective traction force reaction on the scaffolds such that they receive a static tensile force parallel to the constraint direction [107, 108] (Figure 4a). Unlike the passive mechanical stretching force, the direction of cell reorientation is generally aligned with the cell-induced static tensile force [19] (Figure 4c). This static stretch mode seems to be more effective for vessel alignment. Krishnan *et al*. [105] compared the growth of rat microvessel fragments in a 3D collagen gel exposed to a static external load, cyclic external load and anchored boundary conditions. In the anchored group, the internal traction force led to the alignment of the collagen fibrils, and the cells aligned with the fibrils via contact guidance and generated traction force along the direction of the fibrils; this process generated a positive feedback loop. Anchoring also had the greatest effect on neovessel orientation, whereas static and cyclic mechanical stretching did not cause a significant change in orientation compared with anchoring alone. Under similar anchored boundary constraint conditions, Rosenfeld *et al*. [19] applied different inhibitors to change the degree of cell-induced contractile forces and found that the force intensity was positively correlated with vessel elongation.

Boundary conditions and supporting cells are key factors in achieving an appropriate static tensile force [19, 109]. One study showed that under the same anchored boundary conditions, the static tensile force in fibrin gel embedded with cocultures of ECs and fibroblasts was approximately four times greater than that in fibrin gel containing only ECs [19]. In a 3D hexahedral vascularized construct in which constraints were implemented along the long axis, microvessels were aligned parallel to the constrained axis after a few days. When constraints were implemented along the short axis, the orientation of vessels was random because the tensile force quickly dissipated before it provided any meaningful directional guidance to the growing neovessels [108]. By applying 3D bioprinting technology, Zhang *et al*. [36] anchored the ends of hydrogel strips laden with HUVECs and fibroblasts to a polycaprolactone frame. They successfully produced functional blood vessels in the direction of static stretch with no branches, and they found that the regulation of vascular formation by contractile force was mediated by *Yes-associated protein (*YAP*)/transcriptional coactivator with a PDZ-binding domain (*TAZ*)* signalling.

##### Shear stress and dynamic flow culture

Shear stress also contributes to vessel formation [110]. Due to the friction between the inner surface of blood vessels and blood flow, ECs and pericytes are continuously exposed to a tangential force called shear stress [21, 95, 111]. ECs directly sense flow shear stress on their apical sides through a large number of mechanosensors (e.g. cation channels, glycocalyx, primary cilium, G-protein coupled receptors and junctional proteins) and actively respond to induced mechanotransduction signals [112, 113]. Studies have shown that shear stress regulates various vascular functions, including angiogenic sprouting, lumen formation and vascular homeostasis, which are related to expression of the *Krüppel-like factor 2 (*KLF2*), Nuclear factor-kappa B (*NFκB*), Uncoupling protein 2 (*UCP2*)* and *YAP* genes in ECs [114–118]. Such regulations reflect differences in flow patterns. High laminar shear flow inhibits the proliferation and migration of ECs and stabilizes vessels, whereas low shear flow or disturbed flow increases EC proliferation and inflammatory responses [116, 119, 120]. Shear stress also promotes rearrangement of the cytoskeleton and cell polarization, and thus shear stress is conducive to the arrangement of vessels. ECs exposed to shear stress *in vitro* exhibit a spindle-like morphology and gradually elongate, and the long axis of the cells tends to be consistent with the direction of the shear stress [121, 122]. These effects are also influenced by the strength and duration of shear stress. Ko *et al*. [37] measured the transverse in-plane elasticities of perfusion-cultured endothelium and found that the transverse elasticity of the endothelium in the direction parallel to the perfusion-culture flow was ~70% greater than that in the direction perpendicular to the flow. This finding indicates the effects of shear stress on the elastic anisotropy of ECs. In addition, shear stress plays a key role in promoting the differentiation of stem cells (e.g. mesenchymal stem cells, endothelial progenitor cells and embryonic stem cells) into ECs and modulating the supporting cells around vessels [123–125].

Dynamic flow culture is an effective method for applying shear forces to tissue engineering *in vitro* while ensuring a constant supply of nutrients and required bioactive substances (e.g. proangiogenic growth factors) [3, 126–129]. Using a flow bioreactor, Zohar *et al*. [3] exposed polymeric scaffolds cocultured with HAMECs and fibroblasts to direct flow. Compared with static-cultured constructs, the low constant flow of 0.1 ml/min (with an estimated shear stress of 0.075 Pa) significantly increased the distribution depth of the vessels and ECM and enhanced vessel maturation and stability. Recently, with the development of biofabrication and microfluidic technologies, shear stress applied to the interior of scaffolds has become computable and controllable [130–132]. Born *et al*. [133] constructed a mini-scale bioreactor that provides a highly homogenous flow speed, pressure and shear stress and placed a collagen-based scaffold seeded with stromal vascular fraction cells into the bioreactor. This bioreactor improved cell maintenance, promoted the expression of angiogenic genes and allowed the generation of engineered angiogenic tissue. Flow shear stress is more applicable to scaffolds with patterned hollow channels because it effectively promotes the formation and maturation of vessel-like structures in these channels [134, 135]. Li *et al*. [136] fabricated a 3D microfluidic microchannel network and seeded HUVECs on the channel walls through slow flow. They accurately estimated the wall shear stress distribution in the microchannel network using numerical simulation and successfully cultured confluent microvessels with well-maintained endothelial restrictive barrier functions and cellular responses.

Overall, direct external mechanical strategies simulate the pressure and shear forces exerted on the vascular network by blood flow *in vivo*. They can effectively promote the formation and maturation of preformed vascular networks in tissue engineering scaffolds and control their orientation. However, these strategies have certain limitations. First, their impact on vascularization depends on the specific conditions and time requirements for *in vitro* cultivation, which may pose challenges for future clinical translation. Second, external forces acting on the scaffold may affect various performance aspects, such as deformation of the scaffold under tensile forces, which would lead to decreased pore size, thereby restricting the space for vascular formation. Additionally, improper external forces on tissue-engineered constructs containing functional cells may result in morphological changes and functional impairment of these cells. Excessive shear forces can also cause cells to detach from the scaffold surface, which would reduce the cell density on the scaffold and affect the formation and quality of the new tissue.

#### Indirect external mechanical strategies

##### Electricity

**Figure 5:**
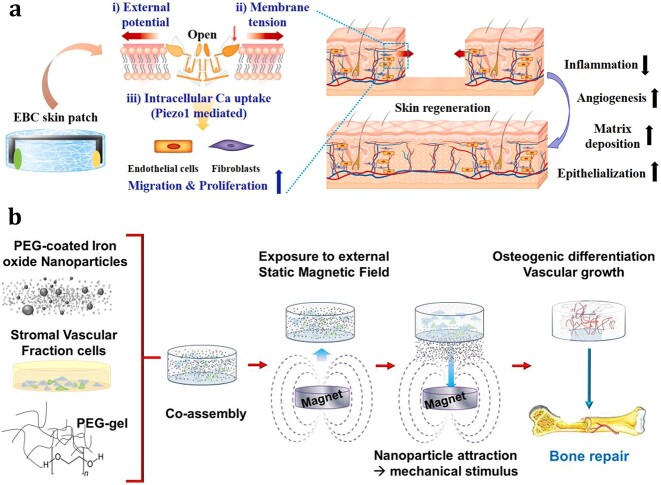
Application of indirect mechanical stimulation to promote vascularization. (**a**) External electrical potential generated by electrical autogenerated skin patches induced membrane tension, which activated Piezo1 channel opening and calcium influx; this in turn stimulated the viability and migration of both ECs and fibroblasts, promoted angiogenesis and accelerated wound healing. Reprinted from Kim *et al*. [138] (Copyright 2021 Elsevier Ltd.) (**b**) MNPs were incorporated into hydrogel scaffolds containing stromal vascular fraction cells from human adipose tissue. After exposure to a static magnetic field, the MNPs migrated freely through and out of the material following the magnetic gradient, which stimulated vascular growth and osteogenic differentiation. Reprinted from Filippi *et al*. [142] (Copyright 2019 Elsevier Ltd.). *ECs* endothelial cells,* EBC* enzymatic-biofuel-cell, *MNPs* magnetic nanoparticles, *PEG* polyethylene glycol

The mechanical and electrical signals of cells are interrelated. Among these signals, potential changes in cell membranes have been demonstrated to directly alter the volume and membrane tension of cells and activate the mechanosensitive ion channel Piezo1. The activation of Piezo1 influences the proliferation, migration and alignment of ECs and participates in the stabilization and maturation of blood vessels [137–139]. Moreover, one study indicated that the application of direct current electric fields can direct the migration and proliferation of ECs and upregulate important chemokine receptors, such as CXCR4 and CXCR2, which play roles in angiogenesis and wound healing [140]. ECs from different sources appear to respond differently to electrical stimulation. Research has shown that ECs derived from angiogenic microvascular tissues, as opposed to nonangiogenic macrovascular tissues, exhibit a more robust response to electrical stimulation [141]. Using an electricity autogenerating skin patch, Kim *et al*. [138] manufactured an external electrical potential that induced sufficient membrane tension in ECs and stimulated the viability and migration of ECs *in vitro*. This skin patch was also shown to enhance blood vessel formation *in vivo* (Figure 5a).

##### Magnetism

The combination of external magnetic fields and magnetic nanoparticles (MNPs) in these fields enables the remote actuation of magnetic force in tissue engineering [143,144]. External magnetic fields manipulate the distribution of MNPs within the 3D space of scaffolds, thus providing the necessary magneto/mechanical stimulation to cells in scaffolds [145,146]. These magneto/mechanical stimuli can also be used to promote vascularization. Filippi *et al*. [142] incorporated MNPs into a hydrogel scaffold containing stromal vascular fraction cells from human adipose tissue. Through magnetic actuation by an external static magnetic field, the VEGF level and endothelial proliferation increased, which resulted in the formation of more elongated capillary-like structures (Figure 5b). Shou *et al*. [147] developed a hydrogel containing magnetic microparticles, fibroblasts and keratinocytes for diabetic wounds. External magnetic fields activated the fibroblasts in these hydrogels, enhanced their proliferation and collagen deposition capabilities, and promoted angiogenesis by improving the paracrine characteristics of the keratinocytes. Luo *et al*. [144] exploited the kinetic properties of MNPs in a magnetic field and constructed a novel artificial bone scaffold loaded with superparamagnetic plasmid gene microspheres. In the presence of a magnetic field, the magnetic microspheres generated magnetic micromovement, which promoted the release of plasmid genes from the microspheres for the transfection of surrounding cells, resulting in VEGF protein expression and angiogenesis promotion.

##### Ultrasound

Ultrasound is a commonly used diagnostic and therapeutic tool in clinical practice. This technique transmits mechanical and thermal energy to deep tissues in the form of waves. Low-intensity pulsed ultrasound (LIPUS), in which minimal thermal effects are produced while maintaining the transmission of acoustic energy, serves as a noninvasive physical stimulus for therapeutic applications [148, 149]. *In vitro*, the exposure of ECs to LIPUS effectively promoted angiogenesis and vascular remodelling, which are associated with activation of the YAP/TAZ and v-akt murine thymoma viral oncogene homologue (AKT) signalling pathways [150, 151]. As a source of remote mechanical stimulation, ultrasound has been progressively introduced to tissue engineering and is used to promote vascularization [152, 153]. By applying travelling ultrasound to a collagen gel embedded with HUVECs, Imashiro *et al*. [154] reported that ultrasound can enhance the interconnections of ECs and promote the formation of vascular networks in a 3D environment. Kang *et al*. [155] exposed porous collagen scaffolds cocultured with HUVECs and adipose-derived stem cells to LIPUS. The results showed that, after LIPUS, cell growth on the collagen scaffold was enhanced ~1.85-fold compared with the control, a difference that was significant. The authors further implanted the scaffolds into rat subcutaneous tissue and continued the LIPUS treatment. Compared with scaffolds that received no such treatment, scaffolds that received LIPUS exhibited more effective angiogenesis, as evidenced by significantly increased expression of specific EC markers such as platelet endothelial cell adhesion molecule-1 (CD31) and vascular endothelial cadherin [155].

In summary, certain indirect external mechanical strategies, including electrical and magnetic stimulation in addition to sound waves, can transmit mechanical stress to cells within scaffolds without direct contact, effectively promoting vascularization [141,142,155]. Due to their remote controllability, the use of these strategies can achieve sustained stimulation after implantation *in vivo*. However, to date, these indirect external mechanical strategies have mainly been used as overall stimuli for the scaffold, in which it is difficult to control the direction of the vascular networks. Additionally, the biological safety of materials such as MNPs and bioelectrodes, which need to be implanted directly with the scaffold into the body, must be carefully considered.

## Conclusions

The mechanical microenvironment plays a significant regulatory role in the formation and alignment of preformed vascular networks in engineered tissues, as well as in the ingrowth of host blood vessels after implantation. Currently, with the continuous exploration of vasculogenesis and angiogenesis mechanisms *in vivo* as well as advancements in bioprinting and microfabrication techniques, the optimization of the mechanical environment in tissue engineering is trending towards precise, dynamic and personalized attributes. Various mechanical strategies effectively promote vascularization in tissue engineering by optimizing the mechanical environment within engineered tissue. Among them, optimizing the mechanical properties of scaffolds, including controlling scaffold stiffness, increasing surface roughness and anisotropic structure, and designing interconnected, hierarchical pore structures, is beneficial for the *in vitro* formation of vascular networks and the ingrowth of host blood vessels. Incorporating hollow channels into scaffolds promotes the formation of patterned vascular networks and guides the ingrowth of host blood vessels. Dynamic stretching and perfusion can facilitate the formation and maturation of preformed vascular networks *in vitro*. Several indirect mechanical strategies can provide sustained mechanical stimulation to engineered tissues *in vivo*, which further promotes the vascularization of implants within the body. Additionally, the role of mechanical strategies in controlling the preformed alignment of vascular networks *in vitro* is significant. Stiffness gradients, anisotropic substrates and hollow channels in scaffolds, as well as external cyclic stretch, boundary constraints and dynamic flow culture, can all regulate the alignment of vascular networks, thereby promoting better integration of prevascularized engineered tissues with host blood vessels upon implantation. With the continuous optimization of mechanical environments and the combination of other chemical and biological factors, it is believed that the challenge of vascularization in tissue engineering will gradually be overcome.

## Abbreviations

ECs: Endothelial cells; ECM: Extracellular matrix; HUVECs: Human umbilical vein ECs; VEGF: Vascular endothelial growth factor; HAMECs: Human adipose microvascular ECs; YAP: Yes-associated protein; TAZ: Transcriptional coactivator with a PDZ-binding domain; CXCR: CXC-chemokine receptor; MNPs: Magnetic nanoparticles; LIPUS: Low-intensity pulsed ultrasound.
